# Identification of Ligand-Responsive RNA G-Quadruplexes in the 3′ UTRs of Dengue Virus Serotypes

**DOI:** 10.3390/biom16070946

**Published:** 2026-06-25

**Authors:** Mohammad Jafar Sheikhi, Ayuka Onuma, Yutaro Imachi, Akira Shiraishi, Shoko Mori, Kohtaro Sugahara, Daisuke Miyoshi, Yue Ma, Takayuki Hishiki, Kazuo Nagasawa, Masayuki Tera

**Affiliations:** 1 Department of Biotechnology and Life Science, Faculty of Engineering, Tokyo University of Agriculture and Technology, 2-24-16, Naka-cho, Tokyo 184-8588, Japan; fy6655@go.tuat.ac.jp (M.J.S.); s263639v@st.go.tuat.ac.jp (Y.I.);; 2 Bioorganic Research Institute, Suntory Foundation for Life Sciences, 8-1-1 Seikadai, Seika-cho, Soraku-gun, Kyoto 619-0284, Japan; 3 Faculty of Frontiers of Innovative Research in Science and Technology (FIRST), Konan University, Kobe 650-0047, Japan; 4 Laboratory for Biomaterials and Bioengineering, Institute of Science Tokyo, 2-3-10 Kanda-Surugadai, Chiyoda-ku, Tokyo 101-0062, Japan; 5 Department of Drug Development, National Institute of Infectious Diseases, Japan Institute for Health Security, Tokyo 162-8655, Japan

**Keywords:** dengue virus, 3′ untranslated region, potential quadruplex-forming sequence, RNA G-quadruplexes

## Abstract

Dengue virus (DENV), which comprises four antigenically distinct serotypes (DENV-1 to DENV-4), remains a major global public health concern and continues to expand geographically; however, the structural features of the viral genome remain incompletely understood. Although G-quadruplexes (G4s) have previously been reported in coding regions of DENV, their presence within the 3′ untranslated region (3′ UTR) has not been experimentally characterized. Here, we focused on selected guanine-rich motifs within the 3′ UTRs of DENV-1 to DENV-4 and investigated their ability to form RNA G4 structures. Using bioinformatic analysis, we identified comparable G-rich regions in the 3′ UTRs of the four serotypes, with serotype-dependent differences in conservation. We then examined the propensity of the selected putative quadruplex-forming sequences (PQSs) to adopt G4 structures using circular dichroism spectroscopy, UV melting analysis, ^1^H NMR spectroscopy, ligand-binding analysis, and reverse transcription stop (RT-stop) assays. Our results provided in vitro evidence that the 3′ UTR oligonucleotides from DENV-1 to DENV-4 are capable of forming ligand-responsive G4 structures, with serotype-dependent differences in conservation, stability, and conformational homogeneity. In addition, reverse transcription (RT)-stop analysis revealed ligand-dependent arrest at the corresponding PQS sites in the presence of the G4 ligand 6OTD, which stabilizes G4 structures. These findings suggest the DENV 3′ UTR as an additional source of ligand-responsive RNA G4-forming elements and support future studies on their possible roles in DENV RNA regulation.

## 1. Introduction

Mosquito-borne viruses (MBVs) constitute a major threat to global public health [1,2]. Their impact has increased rapidly in recent decades because of climate change, population growth, global travel and trade, and the continued expansion of mosquito vectors, which have facilitated transmission in both endemic and newly affected regions [1]. Among important MBVs affecting humans, dengue virus (DENV) is the most widespread mosquito-borne viral infection in humans and remains a major public health concern, with sustained transmission across tropical and subtropical regions and continued spread into newly affected areas, including parts of Europe and the Eastern Mediterranean [3,4,5].

DENV, a member of the family *Flaviviridae* and the genus *Orthoflavivirus*, is classified into four antigenically distinct serotypes, DENV-1 to DENV-4, and possesses a single-stranded, positive-sense RNA genome of approximately 11 kb [6,7,8]. The DENV genome consists of a 5′-capped untranslated region, a single open reading frame encoding a polyprotein that is cleaved into three structural proteins (C, prM, and E) and seven non-structural proteins (NS1, NS2A, NS2B, NS3, NS4A, NS4B, and NS5), and a 3′ UTR that plays essential roles in viral replication and RNA stability [7,8].

Although infections caused by DENV-1 to DENV-4 can show broadly similar clinical features, the four serotypes are phylogenetically distinct and differ in genomic diversity and geographic circulation patterns [9]. Additional variation also occurs within individual serotypes, as reported for DENV-2 genotypes with differences in viral fitness and pathogenic potential [10]. Such diversity may influence viral transmission, epidemiological patterns, disease outcome, and the development of effective control strategies [9,10].

DENV vaccine development remains challenging because of the complex biology and epidemiology of the virus, particularly the presence of four antigenically distinct serotypes [10,11]. In addition, waning immunity and declining neutralizing antibody levels may increase the risk of antibody-dependent enhancement (ADE), highlighting the limitations of vaccine-based approaches and further complicating long-term protection [12]. Moreover, no broadly approved specific antiviral treatment is currently available, underscoring the need for alternative therapeutic strategies [13,14]. Although antiviral approaches have mainly focused on viral proteins, the limited number of DENV-encoded proteins restricts the range of available targets [13].

Such limitation has increased interest in viral RNA structures, particularly conserved structural elements within the viral genome that may influence RNA folding, stability, or interactions with viral and host factors [15,16]. For this reason, analyses using short RNA oligonucleotides provide a useful starting point for structural characterization and can help guide future studies in full-length viral RNA or infection-based systems [17]. Among these, G-quadruplexes (G4s) are non-canonical nucleic acid structures formed by guanine-rich DNA or RNA sequences [18,19]. G4s are formed when guanine-rich nucleic acid sequences fold into stacked planar layers known as G-quartets [20,21]. In each G-quartet, four guanine bases are connected through Hoogsteen hydrogen bonding, and the stacking of multiple quartets generates a compact four-stranded structure [20,21]. These structures are further stabilized by monovalent cations, particularly K^+^, which coordinate within the central channel between stacked G-quarters [20,21]. Depending on strand orientation and loop arrangement, G4s can adopt different topologies, including parallel, antiparallel, and hybrid conformations, although RNA G4s commonly favor parallel arrangements [20,21,22]. Because G4 formation could potentially alter local nucleic acid folding and affect interactions with proteins or ligands, G4s have been linked to important biological processes, including transcription, translation, replication, and RNA stability [18,21,23]. In viral genomes, G4s are increasingly recognized as functionally relevant RNA elements that influence multiple stages of the viral life cycle [24,25,26].

Previous studies have identified G4 structures in the coding regions of the DENV genome, including a G4-forming motif in NS5 that is shared among all four serotypes [27]. Interestingly, G4 structures have been reported in the 3′ UTRs of several other flaviviruses, including Zika virus (ZIKV) and tick-borne encephalitis virus, suggesting that guanine-rich RNA structures in flaviviral 3′ UTRs may be functionally relevant [26,28,29,30,31]. The flaviviral 3′ UTR is a key regulatory region that contains conserved RNA structural elements involved in viral RNA replication, genome stability, long-range 5′–3′ RNA interactions, and subgenomic flaviviral RNA production [8,32,33]. However, G4-forming motifs in the DENV 3′ UTR remain less well characterized. In the present study, we combined bioinformatic, biophysical, and biochemical analysis to determine whether selected PQSs within the 3′ UTRs of DENV-1 to DENV-4 adopt G4 structures in vitro and can be stabilized by the G4 ligand 6OTD, a hexaoxazole telomestatin derivative, to induce reverse transcription arrest.

## 2. Materials and Methods

### 2.1. Identification of PQS Motifs in DENV Genomes

Complete genome sequences representing the four DENV serotypes were retrieved from the NCBI database (National Center for Biotechnology Information, Bethesda, MD, USA) under the following accession numbers: AB178040 (DENV-1 strain NIID02-20); LC367234.1 (DENV-2 strain D2/Hu/India/NIID74/2009); KU050695.1 (DENV-3 strain H87); and LC069810.1 (DENV-4 strain D4/Hu/India/NIID48/2009). Putative G-quadruplex-forming sequences (PQSs) were identified using two independent tools: QGRS Mapper (Ramapo College of New Jersey, Mahwah, NJ, USA; https://bioinformatics.ramapo.edu/QGRS/analyze.php, accessed on 27 March 2026) and PQSfinder (Masaryk University, Brno, Czech Republic; https://pqsfinder.fi.muni.cz/, accessed on 1 June 2026) [34,35]. Each 3′ UTR sequence was analyzed individually to screen for G-rich motifs with the potential to form G4 structures.

### 2.2. DENV 3′ UTR Sequence Collection and Multiple Sequence Alignment Analysis

DENV-1 (*n* = 1312), DENV-2 (*n* = 903), DENV-3 (*n* = 690), and DENV-4 (*n* = 137) complete genome sequences were retrieved from the NCBI Virus database, and only sequences annotated as complete genomes from human hosts (Homo sapiens) were included. Records lacking an annotated complete 3′ UTR were excluded. The 3′ UTR sequences were extracted from GenBank records based on annotated feature coordinates using a custom Python script (Python 3.11.1; Python Software Foundation, Wilmington, DE, USA) implemented in BioPython (Biopython 1.80; Biopython Project; https://biopython.org/), grouped by serotype, and exported as separate FASTA files for downstream analysis. Multiple sequence alignments were then performed separately for each serotype using MAFFT version 7 (Osaka University, Osaka, Japan) in Geneious Prime version 2026.1 (Biomatters Ltd., Auckland, New Zealand), with the default settings [36]. Nucleotide frequencies at each aligned position were calculated, and sequence logos were generated using WebLogo 3 server (University of California, Berkeley, CA, USA; https://weblogo.threeplusone.com/, accessed on 29 March 2026) [37] to assess the conservation and positional distribution of G-rich motifs within the DENV 3′ UTR.

### 2.3. Chemicals

All chemicals were purchased from Fujifilm Wako Pure Chemical Corporation (Osaka, Japan), unless otherwise specified. L2H2-6OTD (6OTD) and 6OTD-Np were synthesized as previously reported [38,39]. Fluorescence measurements were performed using a Spark plate reader (Tecan Group Ltd., Männedorf, Switzerland) in black 384-well flat-bottom non-binding microplates (Greiner Bio-One International GmbH, Kremsmünster, Austria). Each condition was measured in triplicate, and data were analyzed using GraphPad Prism 9.2.9 (GraphPad Software, Boston, MA, USA).

### 2.4. Oligonucleotides

Wild-type and mutant RNA oligonucleotides were obtained from Hokkaido System Science Co., Ltd. (Sapporo, Hokkaido, Japan) as high-performance liquid chromatography (HPLC)-purified, lyophilized products. The mutant RNA oligonucleotides listed in Table 1 were designed by substituting selected guanine residues within the predicted G4-forming motifs with adenine residues to disrupt G4 folding and serve as negative controls. A previously reported severe acute respiratory syndrome coronavirus 2 (SARS-CoV-2) SC-2 RNA G4-forming sequence was used as a positive control representing a known two-layer RNA G4 [40]. The oligonucleotides were dissolved in nuclease-free water (Nacalai Tesque, Kyoto, Japan) to final concentrations of 100 or 200 µM. RNA concentrations were determined using a NanoDrop 1000 spectrophotometer (Thermo Fisher Scientific, Waltham, MA, USA). DNA oligonucleotides and primers were synthesized by Eurofins Genomics K.K. (Tokyo, Japan) in salt-free or OPC-purified grade and supplied pre-dissolved in nuclease-free water at concentrations of 50 µM or 100 µM.

### 2.5. Circular Dichroism (CD) Spectroscopy

For CD measurements, wild-type and mutant RNA oligonucleotide stock solutions were diluted to a final concentration of 2 µM in 10 mM lithium cacodylate buffer (pH 7.4). Measurements were performed under two ionic conditions: a potassium-containing buffer consisting of 10 mM lithium cacodylate buffer (pH 7.4) supplemented with 100 mM KCl, and a potassium-free control buffer consisting of 10 mM lithium cacodylate buffer (pH 7.4) containing 100 mM LiCl instead of KCl. CD spectra were recorded on a J-720 spectropolarimeter (JASCO Corporation, Tokyo, Japan) using a quartz cell with a 1 mm path length, a scanning speed of 500 nm min^−1^, a response time of 1 s, and a wavelength range of 230–320 nm. For each spectroscopic measurement, spectra were recorded three times, and the mean value was used for subsequent analysis. Measurements were carried out at 25 °C, and the corresponding buffer spectrum was used as the baseline.

### 2.6. UV Melting and Thermal Differential Spectra (TDS) Analysis

Wild-type and mutant RNA oligonucleotide stock solutions were prepared in 20 mM potassium phosphate buffer (pH 7.4) containing 70 mM KCl and 10% D_2_O and diluted with the same buffer to a final concentration of 10 µM. After annealing at 95 °C for 5 min and slow cooling to room temperature, UV measurements were performed on a V-730 UV/Visible spectrophotometer (JASCO Corporation, Tokyo, Japan) equipped with a water-cooled Peltier cell changer (PAC-743) (JASCO Corporation, Tokyo, Japan), using quartz cuvettes with a 10 mm optical path length. For each spectroscopic measurement, spectra were recorded three times, and the mean value was used for subsequent analysis. Absorbance at 295 nm was monitored during heating from 25 to 85 °C at a rate of 1 °C/min. Melting curves were analyzed using the baseline method [41]. Briefly, linear baselines were fitted to the low- and high-temperature regions of each curve and used for baseline correction across the full temperature range. The absorbance at 295 nm was then baseline-corrected and normalized, and *T*_m_ was defined as the temperature at which the normalized transition fraction was 0.5. For TDS analysis, UV absorbance spectra were recorded from 280 to 320 nm at low and high temperatures selected according to the thermal stability of each RNA sequence, with a scan speed of 1000 nm/min and a data interval of 1 nm. The temperature was maintained using a thermostatic circulating water bath for the cell holders. The TDS profile was generated by subtracting the spectrum recorded at low temperature from that recorded at high temperature, representing the spectral difference between the folded and unfolded states.

### 2.7. ^1^H NMR Analysis

RNA oligonucleotides were dissolved to a final concentration of 100 µM in potassium phosphate buffer (20 mM, pH 7.4) containing 70 mM KCl and 10% deuterium oxide (D_2_O). The samples were annealed at 95 °C for 5 min and slowly cooled to 25 °C. Water-suppressed 1D ^1^H NMR spectra were recorded at 298 K on a Bruker AVANCE III HD 800 spectrometer equipped with a 5 mm TCI cryogenic probe and a *z*-axis gradient (Bruker BioSpin AG, Fällanden, Switzerland) using 5 mm Shigemi NMR tubes (SHIGEMI Co., Ltd., Tokyo, Japan). Chemical shifts (δ, ppm) were referenced to the residual water signal at δH 4.72 ppm. Spectra were acquired using the standard Bruker pulse sequence zgesgp, zero-filled once, and multiplied by a 4 Hz exponential window function prior to Fourier transformation. Data were processed using TopSpin v3.8.0 (Bruker BioSpin AG, Fällanden, Switzerland).

### 2.8. Fluorescence-Based Ligand-Binding Analysis

For ligand-binding analysis, RNA oligonucleotides were prepared in 20 mM lithium cacodylate buffer containing 100 mM KCl, heated at 95 °C for 5 min, and slowly cooled to room temperature to allow folding. The folded RNA samples were serially diluted to final concentrations ranging from 20 to 0.0375 µM, and 6OTD-Np was added to a final concentration of 0.5 µM. Samples were incubated overnight at room temperature prior to measurement. Fluorescence intensity was recorded at excitation and emission wavelengths of 392 and 602 nm, respectively. The apparent dissociation constant ($K_{d}$) was obtained by fitting the fluorescence titration curves to the following quadratic binding equation:

$$Y=Y_{\min}+a\times \frac{(L_{0}+X+K_{d})-\sqrt{\left(L_{0}+X+K_{d}{)}^{2}-4L_{0}X\right.}}{2L_{0}}$$

where Y  is the fluorescence intensity, $Y_{min}\;$ is the baseline signal, a  is the fluorescence amplitude, X  is the RNA concentration, and $L_{0}$ is the fixed 6OTD-Np concentration.

### 2.9. RT-Stop Assay

To assess G4 formation in DENV PQS motifs and their effect on reverse transcription in the presence of 6OTD, an RT-stop assay was performed. Wild-type PQSs and mutant control sequences designed to disrupt G4 formation were cloned into a custom plasmid backbone containing a T7 promoter, which was synthesized by GenScript Biotech Corporation (Piscataway, NJ, USA), using NEBuilder^®^ HiFi DNA Assembly Master Mix (New England Biolabs, Ipswich, MA, USA). All plasmid constructs were confirmed by Sanger sequencing. The sequences of all wild-type and mutant inserts, as well as the primers used for cloning and sequencing, are provided in (Table S1). The resulting plasmids were amplified by PCR with Phusion™ High-Fidelity DNA Polymerase (New England Biolabs, Ipswich, MA, USA) using the forward primer (5′-AGCTCTTAAGGCTAGAGTACA-3′) and the reverse primer (5′-ACTGAGGCCCAGTGATCATG-3′) to generate linear DNA templates. PCR was carried out with an initial denaturation at 96 °C for 1 min, followed by 25 cycles of 96 °C for 10 s, 63 °C for 20 s, and 72 °C for 10 s, with a final extension at 72 °C for 5 min. The amplified linear DNA products were purified using the NucleoSpin Gel and PCR Clean-up kit (MACHEREY-NAGEL GmbH & Co. KG, Düren, Germany) according to the manufacturer’s protocol.

RNA transcripts were then generated from the linear DNA templates using the ScriptMAX^®^ Thermo T7 Transcription Kit (TOYOBO Co., Ltd., Osaka, Japan) at 40 °C for 4 h. Residual DNA template was removed by treatment with TURBO DNase (Thermo Fisher Scientific, Waltham, MA, USA) at 37 °C for 30 min. The resulting RNA transcripts were further purified using a NucleoSpin purification kit (MACHEREY-NAGEL GmbH & Co. KG, Düren, Germany), diluted to a final concentration of 8 µM in 50 mM Tris-HCl buffer (pH 7.4) containing 100 mM KCl, heated at 95 °C for 5 min, and then slowly cooled to room temperature to allow folding.

For ligand treatment, folded RNA samples (1.5 µM) were incubated overnight at room temperature with 6OTD (0–10 µM) in 1% DMSO.

After overnight incubation, reverse transcription was carried out using PrimeScript™ II Reverse Transcriptase (Takara Bio Inc., Shiga, Japan) with a sequence-specific reverse primer (5′-ACTGAGGCCCAGTGATCATG-3′) which was the same reverse primer used for plasmid PCR amplification. RNA samples were first heated at 65 °C for 5 min and immediately cooled on ice. 6OTD was maintained at the indicated concentration throughout primer annealing and reverse transcription. Reverse transcription was then performed at 50 °C for 45 min, and the enzyme was inactivated by heating at 70 °C for 15 min. Residual RNA was digested with DNase-free ribonuclease glycerol solution (Nippon Gene Co., Ltd., Tokyo, Japan) at 37 °C for 1 h followed by enzyme inactivation by heating at 70 °C for 15 min.

The resulting first-strand cDNA was ligated to a 5′-phosphorylated adaptor (5′-ATTGCCGAGTGACAACTGAA-3′, 3′-AmC7) using ssDNA ligase (New England Biolabs, Ipswich, MA, USA) at 37 °C for 15 min, followed by enzyme inactivation at 95 °C for 5 min. Adaptor-ligated products were then amplified by PCR with Phusion™ High-Fidelity DNA Polymerase (New England Biolabs, Ipswich, MA, USA) under the same cycling conditions described above, using the same sequence-specific primer used for reverse transcription and a reverse primer (5′-TTCAGTTGTCACTCGGCAAT-3′) complementary to the ligated adaptor. PCR products were analyzed by native 8% PAGE at 150 V for 40 min at 4 °C, stained with SYBR Gold (Thermo Fisher Scientific, Waltham, MA, USA) for 10 min, and visualized using a Gel Doc XR imaging system (Bio-Rad Laboratories, Hercules, CA, USA). The bands corresponding to the expected product size were recovered from the gel, purified, and subjected to Sanger sequencing by Eurofins Genomics K.K. (Tokyo, Japan).

### 2.10. Thiazole Orange (TO) Displacement Assay

For the TO displacement assay, RNA oligonucleotides were folded under the same buffer and annealing conditions described for the 6OTD-Np binding assay, and TO was prepared in the same buffer. 6OTD was dissolved in DMSO to obtain a 10 mM stock solution. Folded RNA and TO were mixed at final concentrations of 0.5 and 1.0 µM, respectively, and incubated for 30 min to allow complex formation. 6OTD was then added to final concentrations ranging from 0 to 5.56 µM, followed by overnight incubation at room temperature. Fluorescence was recorded at excitation and emission wavelengths of 500 and 536 nm, respectively. The DC_50_ value was defined as the concentration of 6OTD required to displace 50% of bound TO from the RNA.

## 3. Results

### 3.1. Identification of 3′ UTR Putative G4-Forming Sequences Across DENV Serotypes

To identify guanine-rich motifs with the potential to form G4 structures, one representative complete genome sequence from each of the four DENV serotypes was initially screened using QGRS Mapper and further evaluated using PQSfinder [34]. For the initial PQS screen, one representative complete genome sequence was selected for each serotype, and conservation of the corresponding 3′ UTR region was subsequently assessed across multiple strains by sequence alignment. This subsequent conservation analysis was performed to reduce strain-specific selection bias and to prioritize candidate motifs conserved across multiple strains. Several putative G-quadruplex-forming sequences (PQSs) were identified across the viral genomes, including candidate motifs located in the 3′ UTR region Table 1. Among the 3′ UTR candidates, several PQSs were detected in DENV-1, DENV-2, and DENV-4, whereas only one PQS was identified in DENV-3 using both QGRS Mapper and PQSfinder Tables S2 and S3. The DENV-3 PQS identified by both QGRS Mapper and PQSfinder mapped to a region corresponding to the G-rich motifs observed in the other serotypes, suggesting the presence of a conserved 3′ UTR-associated G4-forming region.

**Figure 1:**
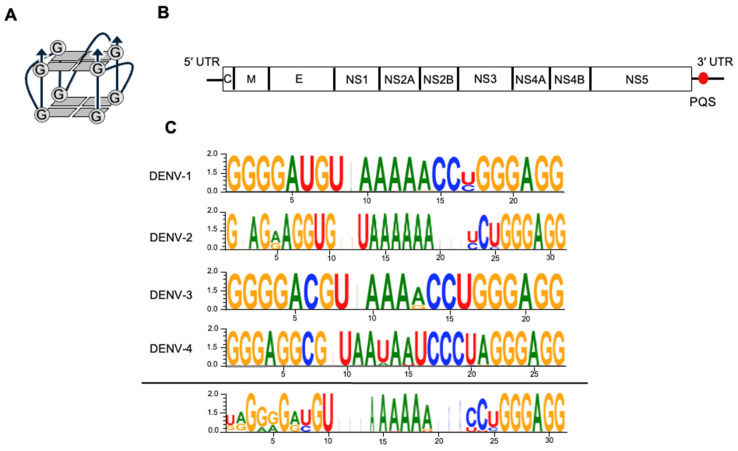
Schematic representation of G4 structure and identification of a conserved G-rich region in the DENV 3′ UTR. (**A**) General schematic representation of RNA parallel G4 structure. (**B**) Schematic of the DENV genome showing the location of the PQS in the 3′ UTR. (**C**) Sequence logos from multiple sequence alignments of the 3′ UTR regions in DENV-1, DENV-2, DENV-3, and DENV-4. Letter height indicates nucleotide conservation, and colors indicate base identity: G, orange; A, green; C, blue; and U, red. Comparable G-rich motifs were observed in all serotypes, with stronger conservation in DENV-1, DENV-3, and DENV-4, whereas DENV-2 showed greater sequence variability. The bottom panel shows the consensus motif.

To further evaluate the conservation of this region, multiple sequence alignment of 3′ UTR sequences from DENV-1 (*n* = 1312), DENV-2 (*n* = 903), DENV-3 (*n* = 690), and DENV-4 (*n* = 137) were performed. The alignment revealed comparable G-rich regions located at broadly corresponding positions within the 3′ UTR of the four serotypes (Figure 1B,C). This region was relatively well conserved in DENV-1, DENV-3, and DENV-4, whereas DENV-2 showed greater sequence variability. The corresponding G-rich motif in DENV-2 was primarily observed in strains belonging to the Cosmopolitan genotype. Based on these findings, this conserved 3′ UTR region was selected for subsequent structural analyses across all four DENV serotypes.

### 3.2. Structural Analysis

**Figure 2:**
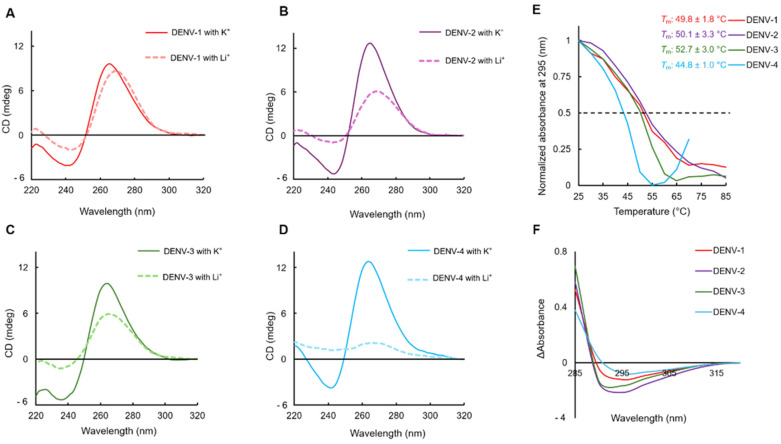
Circular dichroism, UV melting analysis and TDS of DENV G-quadruplex sequences. (**A**–**D**) CD spectra of DENV-1 to DENV-4 wild-type RNA sequences recorded at 25 °C under K^+^ (solid line) and Li^+^ (dashed line): (**A**) DENV-1, (**B**) DENV-2, (**C**) DENV-3, and (**D**) DENV-4. (**E**) UV melting curves monitored at 295 nm for DENV-1 to DENV-4. Apparent *T*_m_ values are presented as mean ± SD from three independent experiments and are indicated for each sequence. (**F**) TDS profile of DENV-1 to DENV-4.

CD spectroscopy was used to assess the folding properties of the four DENV PQSs under potassium-containing conditions and potassium-free LiCl control conditions at 25 °C. In the presence of K^+^, all four wild-type RNA oligonucleotides displayed similar spectral profiles, with a positive band at 265 nm and a negative band near 240 nm (Figure 2A–D). This pattern is consistent with a parallel G4 topology [42], indicating that the PQS motifs from DENV-1 to DENV-4 are capable of folding into parallel RNA G4 structures. Under Li^+^ control conditions, the CD spectra were generally reduced or less well defined compared with the K^+^ condition, supporting the contribution of K^+^ to G4- folding (Figure 2A–D). In contrast, the mutant oligonucleotides showed similar CD profiles under K^+^ and Li^+^ conditions, suggesting that these mutants did not exhibit clear potassium-dependent G4 folding under the same conditions; the mutant-control and SARS-CoV-2 SC-2 positive-control data are shown in (Figure S1). To evaluate their thermal stability, UV melting experiments were performed from 25 °C to 85 °C by monitoring absorbance at 295 nm. All sequences exhibited temperature-dependent changes consistent with G4 unfolding. The melting curves showed temperature-dependent transitions, and apparent *T*_m_ values were estimated to be 49.8 °C for DENV-1, 50.1 °C for DENV-2, 52.7 °C for DENV-3, and 44.8 °C for DENV-4 (Figure 2E). Among the four sequences, DENV-3 showed the highest thermal stability, whereas DENV-4 exhibited the lowest apparent *T*_m_. Based on the apparent melting profiles, sequence-dependent high-temperature conditions were selected for TDS analysis to allow unfolding or partial unfolding of the G4 structures: 85 °C for DENV-1 and DENV-2, 65 °C for DENV-3, and 55 °C for DENV-4. All wild-type DENV 3′ UTR PQS sequences showed a characteristic negative signal with a minimum around 295 nm, consistent with Hoogsteen hydrogen bonding within G-quartets (Figure 2F). In contrast, the negative signal at 295 nm was absent in mutant sequences (Figure S2), indicating that the observed TDS profiles depend on the intact G-rich motifs and are consistent with G4 formation. The SARS-CoV-2 SC-2 G4 sequence was also analyzed as a positive control for UV/TDS analysis, and the corresponding data are shown in (Figure S2). To further assess their G4 formation, ^1^H NMR spectroscopy was performed. Signals in the imino proton region around 10–12.5 ppm are characteristic of guanine imino protons involved in Hoogsteen hydrogen bonding within G-quartets and therefore provide structural evidence for G4 formation [43]. The ^1^H NMR spectra of all four DENV PQS sequences showed imino proton signals consistent with G4 formation (Figure S3). DENV-1, DENV-2, and DENV-3 exhibited broader resonances than DENV-4, suggesting greater conformational heterogeneity on the NMR timescale, whereas DENV-4 showed features consistent with a more homogeneous folded state. The observed spectral broadening may reflect heterogeneous or non-canonical G4-like conformations, including alternative G-register usage, irregular tetrad organization, or multiple folded species.

Taken together, the CD, UV, and ^1^H NMR data support G4 formation by the four DENV PQSs. However, because the NMR analysis was limited to 1D imino proton spectra, detailed strand alignment, strand register, and the possible coexistence of multiple folded conformations could not be fully resolved.

### 3.3. Ligand-Binding Analysis

**Figure 3:**
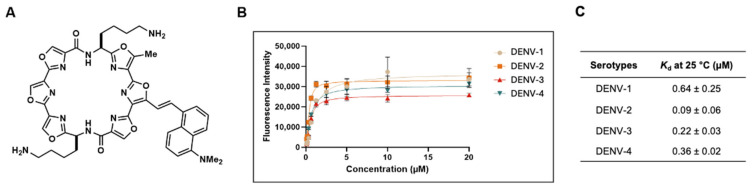
Binding analysis of dengue virus 3′ UTR G4-forming sequences by fluorescence-based ligand-binding assays. (**A**) Chemical structure of 6OTD-Np. (**B**) Fluorescence-based ligand-binding curves for DENV-1 to DENV-4 wild-type RNA sequences (0–20 µM) in the presence of 6OTD-Np (0.5 µM). (**C**) Apparent K_d_ values obtained from 6OTD-Np binding analyses. Data are presented as mean ± SD from three independent experiments.

To further support the formation of ligand-binding G4 structures by the DENV 3′ UTR PQSs, we employed 6OTD-Np, a fluorogenic G4-binding ligand [38,44]. 6OTD-Np is a macrocyclic hexaoxazole telomestatin derivative bearing a rotatable vinylnaphthyl side chain, which becomes conformationally restricted upon binding to a G4 structure, resulting in fluorescence activation [38]. Each DENV RNA PQS was titrated into a solution of 6OTD-Np, and the resulting fluorescence signals were recorded. Fluorescence titration analysis yielded apparent *K*_d_ values in the range of 0.09–0.64 μM (Figure 3B,C), supporting the formation of ligand-binding G4 structures by the selected DENV 3′ UTR sequences in all serotypes. In contrast, the mutant oligonucleotides showed weaker and poorly saturating 6OTD-Np fluorescence responses, indicating reduced ligand interaction compared with the wild-type DENV PQSs (Figure S4). Because reliable apparent *K*_d_ values could not be determined for these weakly saturating mutant curves, these negative controls were used to support binding specificity rather than for direct *K*_d_ comparison.

### 3.4. RT-Stop Assay of DENV 3′ UTR PQSs

G4-binding ligands such as 6OTD are well established to stabilize G4 structures and to induce reverse transcription arrest at folded G4 sites [45,46]. Accordingly, RT-stop assays performed in the presence of G4 ligands have been widely used as a functional readout for G4 formation in nucleic acids. Based on our biophysical and ligand-binding analyses supporting the formation of ligand-responsive G4 structures, we next examined whether these motifs can impede reverse transcription in this assay upon stabilization by 6OTD. To this end, we employed an RT-stop assay. To confirm that 6OTD binds to the G4 structures of DENV-1 to DENV-4, a Thiazole Orange (TO)-displacement assay was performed. The results demonstrated that 6OTD effectively displaced TO from all four G4-forming sequences in a concentration-dependent manner, with DC_50_ values ranging from 0.54 to 0.73 µM, supporting its role as a G4-stabilizing ligand in this assay (Figure S5).

**Figure 4:**
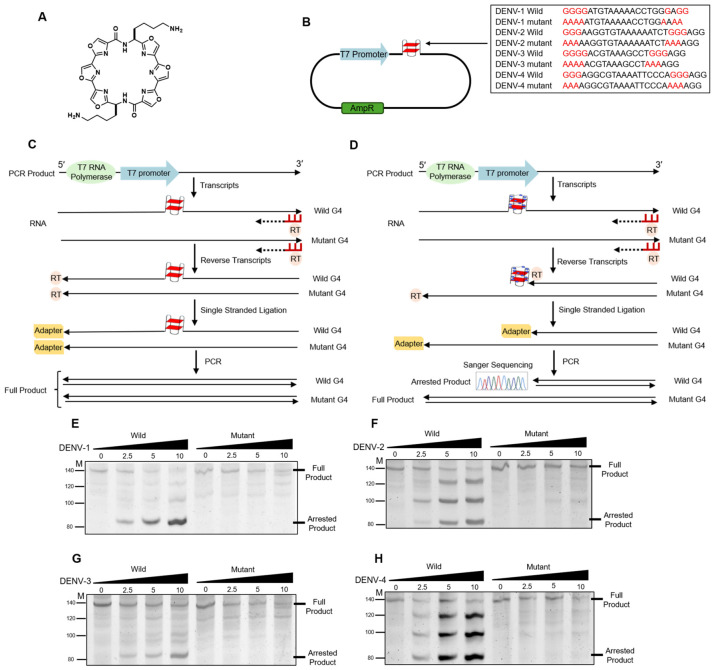
RT-stop analysis of DENV 3′ UTR G4-forming sequences. (**A**) Chemical structure of 6OTD. (**B**) Schematic map of the T7 promoter-containing plasmid construct used to generate RT-stop assay templates and the sequences of the wild-type and mutant DENV-1 to DENV-4 3′ UTR inserts. Red letters indicate guanine residues in the wild-type sequences and the corresponding G-to-A substitutions in the mutant sequences. Colored boxes, arrows, and labels indicate the T7 promoter, T7 RNA polymerase, RT primer, adaptor, and G4 motif, as shown in the schematic diagrams; black lines represent nucleic acid strands or products. All constructs were verified by sequencing. (**C**,**D**) Schematic diagrams of the RT-stop assay workflow in the absence (**C**) or presence (**D**) of ligand-stabilized G4 formation with the purple symbol indicating 6OTD. In the presence of 6OTD, reverse transcriptase is arrested at the wild-type G4 site, generating a truncated cDNA product corresponding to the position of the inserted PQS. (**E**–**H**) Representative native 8% PAGE gels for DENV-1, DENV-2, DENV-3, and DENV-4, respectively. Wild-type and mutant RNAs were analyzed after incubation with increasing concentrations of 6OTD (0, 2.5, 5, and 10 µM). Ligand-dependent RT-stop products were detected specifically in the wild-type templates, whereas mutant RNA templates produced full-length products. Minor higher-molecular-weight arrest bands observed for DENV-2 and DENV-4 arise from an additional downstream G-rich sequence present in the plasmid constructs and are distinct from the intended DENV PQS-derived RT-stop signal. The original images are provided in the Supplementary Materials.

RT-stop analysis was performed using the selected DENV 3′ UTR PQSs in the presence and absence of 6OTD under dose-dependent conditions for both wild-type and mutant G4 sequences (Figure 4). Stabilization of a G4 structure by 6OTD is expected to induce reverse transcription arrest at the inserted G4-forming region, generating truncated cDNA products of defined sizes (Figure 4C,D); gels shown in (Figure 4E–H). At the highest concentration of 6OTD, clear RT-stop bands were detected for all four wild-type DENV sequences, whereas decreasing the 6OTD concentration progressively restored reverse transcription, indicating dose-dependent stabilization of the folded G4 structure. In contrast, mutant sequences designed to disrupt G4 formation showed no RT-stop bands at the corresponding positions even at the highest concentration of 6OTD, and reverse transcription proceeded under all tested conditions. The major RT-stop bands were observed at 84nt for DENV-1 and DENV-3 and at 81nt for DENV-2 and DENV-4, consistent with the different insertion positions of the PQS sequences in the plasmid constructs (Figure 4B–H). In addition, DENV-2 and DENV-4 exhibited additional longer RT-stop bands above the primary arrest site. These bands are attributed to an additional G-rich sequence located downstream of the intended PQS insertion within the plasmid constructs, which is distinct from the analyzed DENV 3′ UTR PQS. Consistent with this interpretation, our local G4 prediction analysis identified an additional potential G-quadruplex-forming site within this downstream region of the DENV-2 and DENV-4 constructs (Figure S6B,D,E) [47]. These additional bands are most likely attributable to the artificial transcript context used in the plasmid-based RT-stop assay and should not be interpreted as additional native DENV 3′ UTR PQS-derived arrest sites. Sanger sequencing of the gel-purified RT-stop products confirmed that the arrest site mapped precisely to the first guanine residue of each wild-type DENV PQS sequence (Figure S6A–D). These results indicate that 6OTD interacts with the folded RNA structures of the selected DENV 3′ UTR PQSs. Together with the RT-stop assay results, these findings indicate that the selected DENV-1 to DENV-4 3′ UTR PQS sequences can form G4 structures that are stabilized by 6OTD, resulting in site-specific reverse transcription arrest.

## 4. Discussion

Our analyses indicate that the DENV 3′ UTR contains comparable G-rich regions capable of forming G4 structures, with serotype-dependent differences in conservation. The motif was relatively well conserved in DENV-1, DENV-3, and DENV-4, whereas DENV-2 showed greater variability, suggesting genotype-dependent maintenance of this putative G4-forming sequence [9,48]. Given that related flaviviruses harbor functional G-rich elements in their 3′ terminal regions, the potential biological relevance of these DENV 3′ UTR G4-forming motifs in viral RNA regulation warrants further investigation [26,28,30,49]. Because this motif lies within a region previously proposed to harbor higher-order RNA structures, G4 formation may coexist with, or modulate, other structured RNA elements in the DENV 3′ UTR; however, the functional significance of this structural interplay remains to be determined [8,50,51,52,53,54,55].

Because the PQSs identified in this study do not conform to typical canonical G4-forming sequences, we comprehensively evaluated their structural properties using three complementary spectroscopic approaches: CD spectroscopy, UV melting/TDS analysis, ^1^H NMR and fluorescence-based analysis with the fluorogenic G4 ligand 6OTD-Np. Taken together, these analyses supported the presence of Hoogsteen hydrogen bonding characteristic of G4 formation, while also revealing serotype-dependent differences in stability and conformational behavior. In particular, the broader and weaker imino proton signals observed for DENV-1, DENV-2, and DENV-3, compared with the more uniform spectrum of DENV-4, suggest greater conformational heterogeneity on the NMR timescale [43,56]. At the same time, the sequence features of these motifs raise the possibility that the folded structures include non-canonical G4 conformations, such as those involving bulges, interrupted G-runs, guanine shifting, alternative guanine usage, or irregular tetrad organization. Notably, the DENV-1 and DENV-3 PQSs contain four consecutive guanine residues at the 5′ end, a feature that may permit alternative G-register usage and loop-less or minimally looped G4 arrangements, as reported for related G-rich sequences [42,56]. Such structural plasticity may contribute to the observed conformational heterogeneity and to the coexistence of multiple folded species [42,56]. The corresponding mutant oligonucleotides provide an additional point of comparison for interpreting these structural features. The altered G4-associated biophysical signatures observed after guanine substitution further support the involvement of the selected G-rich motifs in the observed folding behavior. Accordingly, the DENV-1 and DENV-3 motifs may adopt non-canonical or structurally heterogeneous G4-like conformations despite lacking an obvious canonical G4 arrangement.

6OTD is a well-characterized G4 ligand and was used here as a chemical probe to evaluate whether the selected DENV 3′ UTR PQS motifs adopt ligand-responsive folded structures [39]. The combined fluorescence-based ligand-binding and RT-stop analyses support this interpretation, indicating that these motifs can be recognized and stabilized by a G4-binding ligand such as 6OTD [39,45]. In particular, the site-specific reverse transcription arrest observed in the RT-stop assay, together with sequencing of the arrested products, suggests that ligand-mediated stabilization occurs at defined PQS sites rather than through a nonspecific reduction in reverse transcriptase processivity.

Importantly, G4-binding ligands have previously been reported to suppress viral replication in several viruses, including flaviviruses such as ZIKV, as well as SARS-CoV-2, highlighting viral G4 structures as potential antiviral targets [40,48,57]. Although antiviral activity was not examined in the present study, and 6OTD was used only as an in vitro G4-binding chemical probe rather than as an antiviral drug, the identification of ligand-responsive G4 motifs in the DENV 3′ UTR may provide a useful structural basis for future chemical and functional studies [57,58]. In this context, approaches using longer DENV RNA transcripts, genome-scale analyses, or infectious virus systems will be important to assess whether ligand-stabilized G4 formation occurs in a more biologically relevant setting. In addition, the RT-stop strategy used here could be extended to longer DENV RNA transcripts or genome-scale analyses to systematically examine ligand-stabilized G4 sites in a more native sequence context [45,59].

The additional higher-molecular-weight arrest bands observed for DENV-2 and DENV-4 are also noteworthy. These secondary arrest signals are unlikely to reflect the intended DENV 3′ UTR PQS itself, but rather arise from the artificial plasmid-derived transcript context. Local G4 prediction identified additional downstream G-rich regions in the DENV-2 and DENV-4 constructs, providing a plausible explanation for the second-longest arrest bands (Figure S6). In contrast, the uppermost arrest bands were not readily explained by canonical G4-forming sequences alone and may reflect more noncanonical ligand-responsive G-rich intermediates, such as G-triplex-like conformations [60]. Although further structural analysis will be required to define these species, these additional bands were clearly distinguishable from the primary, site-specific RT-stop signals mapped to the intended DENV 3′ UTR PQS sites.

Since 6OTD is a well-characterized universal G4-stabilizing ligand, the ligand-induced RT-stop assay provides a useful framework for comparing how different RNA regions respond to ligand-mediated stabilization. Overall, the data support the view that the selected DENV 3′ UTR motifs form folded, ligand-responsive G4 structures that can be recognized and stabilized by 6OTD. A limitation of this study is that the structural and ligand-response experiments were performed using simplified RNA models, including short synthetic RNA sequences and in vitro transcript constructs, rather than the complete viral genome in an infection context. Thus, although our data indicate that the selected DENV 3′ UTR motifs can adopt folded, ligand-responsive G4 structures under the experimental conditions tested and can be recognized and stabilized by 6OTD, the biological relevance of these findings during the viral life cycle remains to be established. Further studies using full-length viral RNA and infectious virus systems will be needed to determine whether these structures form and function during DENV infection.

## 5. Conclusions

The present study provides evidence that selected guanine-rich motifs within the 3′ UTRs of DENV-1 to DENV-4 can adopt ligand-responsive RNA G4 structures in vitro and in engineered transcript-based assays. Biophysical analyses supported G4 formation with serotype-dependent differences in stability and conformational behavior, while interaction with 6OTD and RT-stop analysis demonstrated ligand-stabilized reverse transcription arrest at defined PQS sites. Since the present study was performed using oligonucleotides and engineered transcript systems, whether these structures form and function within full-length native viral RNA during DENV infection remains unclear. These findings expand the current understanding of DENV RNA structural architecture and provide a useful basis for future studies investigating the biological roles of 3′ UTR G4-forming motifs during the DENV life cycle.

## Figures and Tables

**Table 1:** Selected putative quadruplex-forming sequences (PQSs) identified in the 3′ UTRs of the four DENV serotypes and their corresponding mutant control sequences. The table shows the reference accession number, genomic position of the selected PQS, and the corresponding wild-type and G-to-A mutant control sequences (5′–3′). Mutant sequences were not predicted by either QGRS Mapper or PQSfinder.

Serotypes	Accession No.	Position of Selected PQS in 3′ UTR	QGRS Mapper Score	PQSfinder Score	WT Selected PQS Sequence (5′–3′)	Mutant Control Sequence (5′–3′)
DENV-1	AB178040.1	10,451–10,472	9	17	GGGGATGTAAAAACCTGGGAGG	AAAAATGTAAAAACCTGGAAAA
DENV-2	LC367234.1	10,434–10,458	11	15	GGGAAGGTGTAAAAAATCTGGGAGG	AAAAAGGTGTAAAAAATCTAAAAGG
DENV-3	KU050695.1	10,414–10,434	10	19	GGGGACGTAAAGCCTGGGAGG	AAAAACGTAAAGCCTAAAAGG
DENV-4	LC069810.1	10,344–10,368	10	18	GGGAGGCGTAAAATTCCCAGGGAGG	AAAAGGCGTAAAATTCCCAAAAAGG

## Data Availability

The data supporting the findings of this study are included within the article and its Supplementary Materials. Additional data are available from the corresponding author upon reasonable request.

## Supplementary Materials

The following supporting information can be downloaded at: https://www.mdpi.com/article/10.3390/biom16070946/s1, Figure S1: CD analysis of mutant DENV 3′ UTR PQSs and SARS-CoV-2 SC-2 positive-control G4 sequence; Figure S2: TDS and UV melting analysis of wild-type and mutant DENV G4 sequences; Figure S3: One-dimensional ^1^H NMR spectra of DENV 3′ UTR PQS sequences; Figure S4: 6OTD-Np fluorescence analysis of DENV 3′ mutant sequences; Figure S5: TO displacement analysis of DENV 3′ UTR PQS RNA sequences; Figure S6: Sequence verification of RT-stop mapping; Table S1: List of primers used for cloning and sequencing; Table S2: Predicted putative G-quadruplex-forming sequences in the dengue virus 3′ UTR identified using QGRS Mapper; Table S3: Putative G-quadruplex-forming sequences identified in the 3′ UTR regions of DENV-1 to DENV-4 using PQSfinder; Original images of Figure 4E–H.

## Abbreviations

The following abbreviations are used in this manuscript:

6OTD	6-oxazole telomestatin derivative
6OTD-Np	6-oxazole telomestatin derivative bearing naphthyl group
cDNA	complementary DNA
ADE	antibody-dependent enhancement
CD	circular dichroism
DC_50_	half-maximal displacement concentration
DENV	dengue virus
DMSO	dimethyl sulfoxide
DNA	deoxyribonucleic acid
D_2_O	deuterium oxide
G4	G-quadruplex
HPLC	high-performance liquid chromatography
K_d_	dissociation constant
MBV	mosquito-borne virus
NCBI	National Center for Biotechnology Information
NMR	nuclear magnetic resonance
nt	nucleotide
OPC	oligonucleotide purification cartridge
PAGE	polyacrylamide gel electrophoresis
PCR	polymerase chain reaction
PQS	putative quadruplex-forming sequence
RNA	ribonucleic acid
RT	reverse transcription
RT-stop	reverse transcription stop
SARS-CoV-2	severe acute respiratory syndrome coronavirus 2
TO	Thiazole Orange
TDS	Thermal Differential Spectra
T_m_	melting temperature
UTR	untranslated region
ZIKV	Zika virus
