# Supplementary material for: Identification of Ligand-Responsive RNA G-Quadruplexes in the 3′ UTRs of Dengue Virus Serotypes
Source: Biomolecules. 2026 Jun 25;16(7):946. doi: 10.3390/biom16070946 (PMC13407133; doi:10.3390/biom16070946)
Supplement: Supplementary file 1 [file biomolecules-16-00946-s001.zip › biomolecules-4325061-proofed supplementary.pdf]

Supporting information for

Identification of Ligand-Responsive RNA G-Quadruplexes in the 3′ UTRs of Dengue Virus Serotypes

Mohammad Jafar Sheikhi[1], Ayuka Onuma[1], Yutaro Imachi[1], Akira Shiraishi[2], Shoko Mori[2], Kohtaro Sugahara[2], Daisuke Miyoshi[3], Yue Ma[4], Takayuki Hishiki[5], Kazuo Nagasawa[1] , Masayuki Tera[1,*]

[1] Department of Biotechnology and Life Science, Faculty of Engineering, Tokyo University of Agriculture and Technology, 2-24-16, Naka-cho, Koganei, Tokyo 184-8588, Japan.
[2] Bioorganic Research Institute, Suntory Foundation for Life Science, 8-1-1 Seikadai, Seika-cho, Soraku, Kyoto 619-0284, Japan.
[3] Faculty of Frontiers of Innovative Research in Science and Technology (FIRST), Konan University, Kobe 650-0047, Hyōgo, Japan.
[4] Laboratory for Biomaterials and Bioengineering, Institute of Science Tokyo, 2-3-10 Kanda-Surugadai, Chiyoda-ku, Tokyo 101-0062, Japan.
[5] Department of Drug Development, National Institute of Infectious Diseases, Japan Institute for Health Security, Tokyo, Japan.
*Correspondence: E-mail: tera@go.tuat.ac.jp

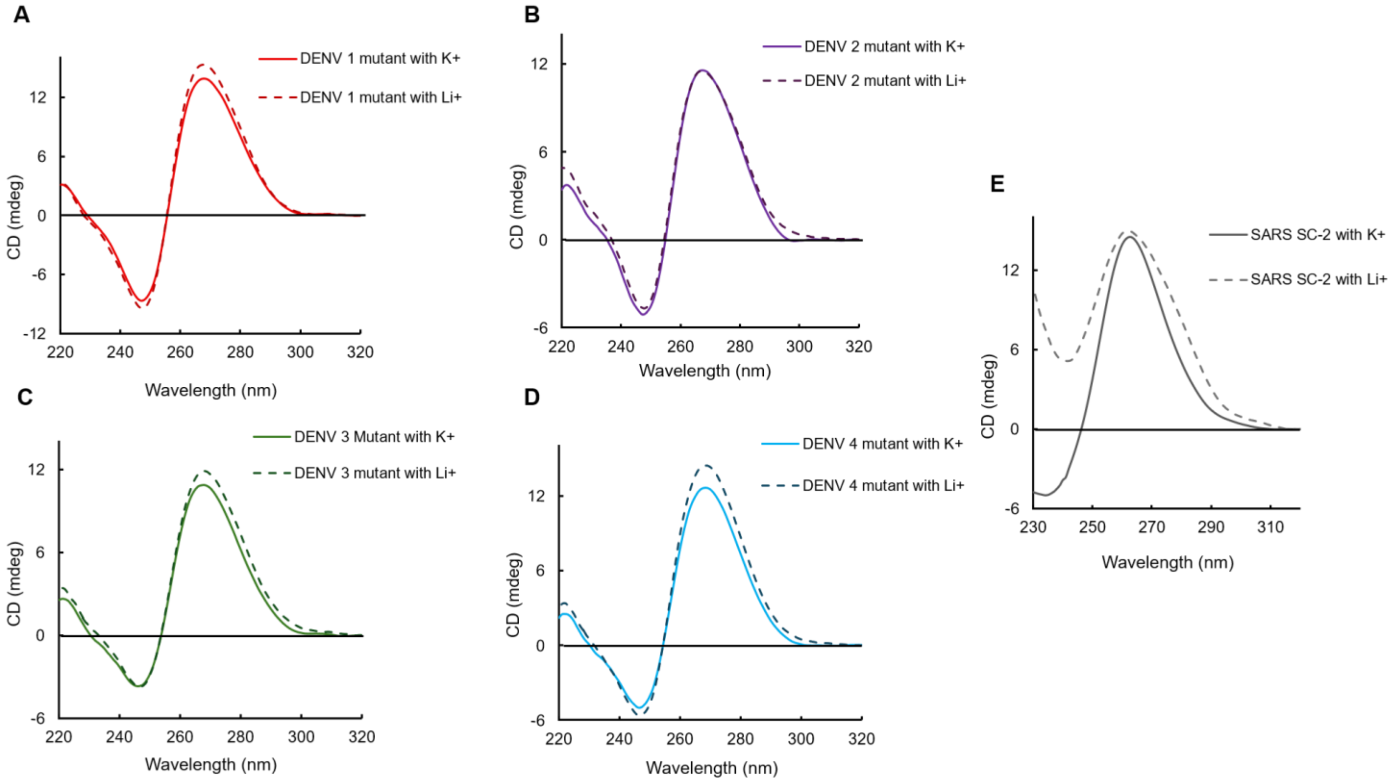

Supplementary Figure S1. CD analysis of mutant DENV 3′ UTR PQS sequences and SARS-CoV-2 SC-2 positive-control G4 sequence. **(A–D)** CD spectra of DENV-1 to DENV-4 mutant RNA sequences recorded under K^+ (solid line) and Li^+ (dashed line) conditions: **(A)** DENV-1, **(B)** DENV-2, **(C)** DENV-3, and **(D)** DENV-4. The mutant sequences showed similar CD profiles under K^+ and Li^+ conditions, suggesting the absence of clear potassium-dependent G4-like folding. **(E)** CD spectra of SARS-CoV-2 SC-2 positive-control sequence recorded under K^+ (solid line) and Li^+ (dashed line) conditions. The SC-2 sequence showed a G4-associated CD signature and was used as a positive control for the CD assay.

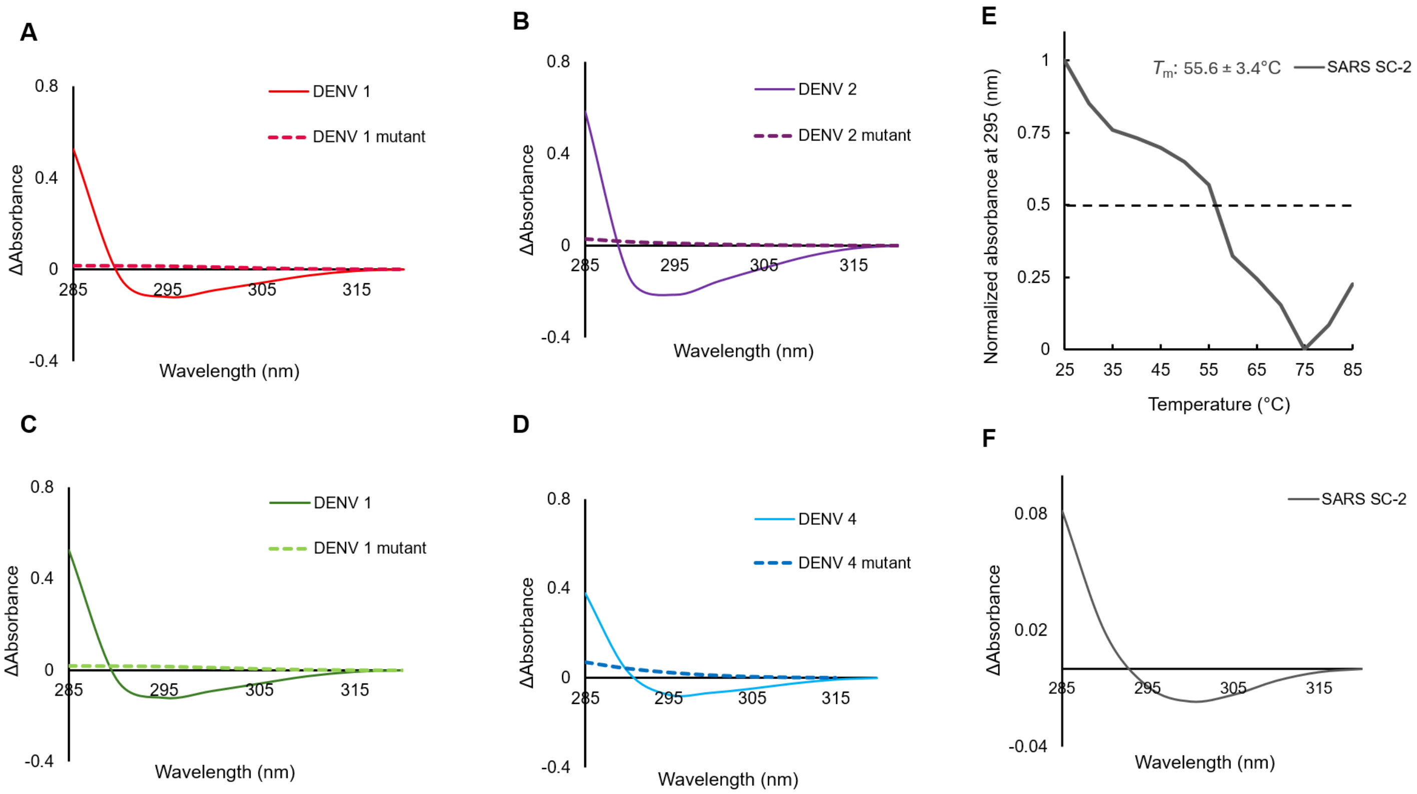

Supplementary Figure S2. TDS analysis of wild-type and mutant DENV G4 sequences. **(A–D)** Thermal difference spectra of DENV-1 to DENV-4 wild-type (solid line) and mutant (dashed line) RNA sequences: **(A)** DENV-1, **(B)** DENV-2, **(C)** DENV-3, and **(D)** DENV-4. Wild-type sequences showed G4-characteristic TDS profiles around 295 nm, whereas mutant sequences showed no signals. **(E)** UV melting curves of SARS-CoV-2 SC-2 positive-control sequence monitored at 295 nm. **(F)** TDS profiles of the SARS-CoV-2 SC-2 positive-control sequence.

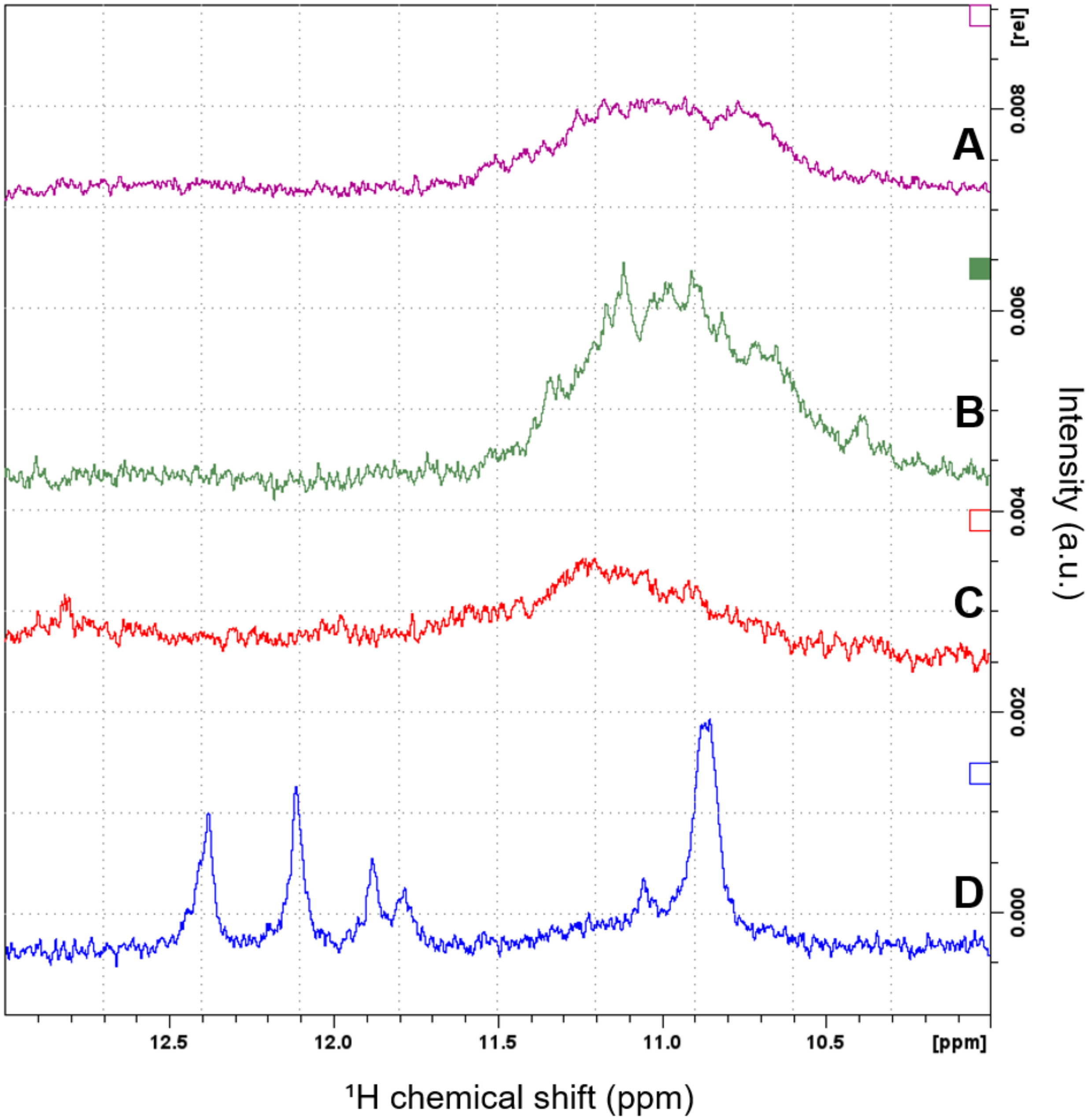

Supplementary Figure S3. ^{1}H NMR spectra of DENV 3′ UTR PQS sequences. **(A–D)** ^{1}H NMR spectra recorded in the imino proton region 10–13 ppm for **(A)** DENV-1, **(B)** DENV-2, **(C)** DENV-3, and **(D)** DENV-4 3′ UTR PQS sequences.

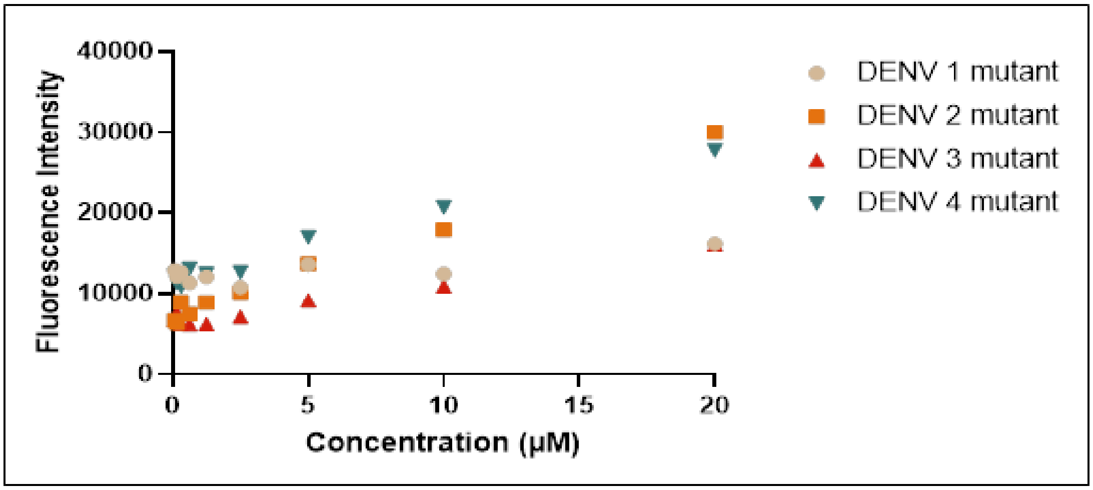

Supplementary Figure S4. 6OTD-Np fluorescence analysis of DENV 3′ mutant sequences. Fluorescence-based ligand binding responses for DENV-1 to DENV-4 mutant RNA sequences (0–20 μM) in the presence of 6OTD-Np (0.5 μM). The mutant sequences showed weak, non-saturating fluorescence responses; therefore, reliable apparent K_d values could not be calculated.

A

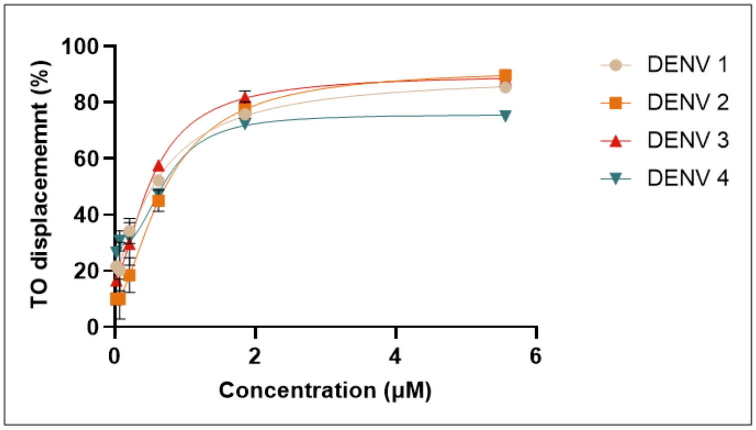

B

**Serotypes**	**DC_{50} (μM)**
DENV 1	0.65 ± 0.013
DENV 2	0.74 ± 0.034
DENV 3	0.54 ± 0.019
DENV 4	0.72 ± 0.019

Supplementary Figure S5. TO displacement analysis of DENV 3′ UTR PQS RNA sequences. **(A)** TO displacement curves for DENV-1 to DENV-4 3′ UTR PQS RNA sequences in the presence of increasing concentrations of 6OTD (0–5.6 μM). **(B)** DC_{50} values obtained from TO displacement analyses. Data are presented as mean ± SD from three independent experiments.

Supplementary Table S1. List of primers used for cloning.

Primer Name		Primer sequence 5'–3'	Application
DENV-1 Wild	Forward	GGGGATGTAAAAACCTGGGAGGTAAAGAATTCAGCTT CCAAGGTGTACG	Cloning
DENV-1 Wild	Revers	TACCTCCCAGGTTTTTACATCCCCAATTAATATGCTAGC CATGGTGGCTC	Cloning
DENV-1 MUT	Forward	AAAAATGTAAAAACCTGGAAAATAAAGAATTCAGCTTC CAAGGTGTACG	Cloning
DENV-1 MUT	Revers	TATTTTCCAGGTTTTTACATTTTTAATTAATATGCTAGCC ATGGTGGCTC	Cloning
DENV-2 Wild	Forward	GGGAAGGTGTAAAAAATCTGGGAGGAATTCAGCTTCC AAGGTGTACG	Cloning
DENV-2 Wild	Revers	CTCCCAGATTTTTTACACCTTCCCTTAATTAATATGCTA GCCATGGTGGCTC	Cloning
DENV-2 MUT	Forward	AAAAAGGTGTAAAAAATCTAAAAGGAATTCAGCTTCCA AGGTGTACG	Cloning
DENV-2 MUT	Revers	CTTTTAGATTTTTTACACCTTTTTTTAATTAATATGCTAG CCATGGTGGCTC	Cloning
DENV-3 Wild	Forward	GGGGACGTAAAGCCTGGGAGGAAGAATTCAGCTTCC AAGGTGTACG	Cloning
DENV-3 Wild	Revers	CCTCCCAGGCTTTACGTCCCC AATTAA TAT GCT AGC CATGGTGGCTC	Cloning
DENV-3 MUT	Forward	AAAAACGTAAAGCCTAAAAGGAAGAATTCAGCTTCCA AGGTGTACG	Cloning
DENV-3 MUT	Revers	CCTTTTAGGCTTTACGTTTTTAATTAATATGCTAGCCAT GGTGGCTC	Cloning
DENV-4 Wild	Forward	GGGAGGCGTAAAATTCCCAGGGAGGAATTCAGCTTC CAAGGTGTACG	Cloning
DENV-4 Wild	Revers	CTCCCTGGGAATTTTACGCCTCCCTTAATTAATATGCTA GCCATGGTGGCTC	Cloning
DENV-4 MUT	Forward	AAAAGGCGTAAAATTCCCAAAAAGGAATTCAGCTTCC AAGGTGTACG	Cloning
DENV-4 MUT	Revers	CTTTTTGGGAATTTTACGCCTTTTTTAATTAATATGCTA GCCATGGTGGCTC	Cloning
Sequencing	Forward	ACTGACATCCACTTTGCCTTTC	Sequencing
Sequencing	Revers	CGTGGCCCACAAAGATGATTTT	Sequencing

Supplementary Table S2. Predicted putative G-quadruplex-forming sequences in the dengue virus 3′ UTR with QGRS Mapper. Putative PQSs identified in the 3′ UTR of representative DENV serotypes. Guanine residues contributing to the predicted motifs are shown in blue and underlined. Selected candidate PQSs for further analysis are highlighted.

Serotype	Position	Length	QGRS	G-Score
DENV-1	10451	22	**GGGGATGTAAAAACCTGGGAGG**	9
	10499	25	GGGGTAGCAGACTAGTGGTTAGAGG	9
	10583	25	GGTGGTAAGGACTAGAGGTTAGAGG	20
DENV-2	10434	25	**GGGAAGGTGTAAAAAATCTGGGAGG**	11
	10485	25	GGCGTAGTGGACTAGCGGTTAGAGG	20
	10541	16	GGGGGCCCAAGGTGGG	17
	10576	21	GGAAGGACTAGAGGTTAGAGG	17
DENV-3	10414	21	**GGGGACGTAAAGCCTGGGAGG**	10
DENV-4	10343	25	**GGGAGGCGTAAAATTCCCAGGGAGG**	10
	10394	25	GGCATATTGGACTAGCGGTTAGAGG	20
	10456	20	GGGGGCCCGAAGCCAGGAGG	12
	10489	24	GGTGGAAGGACTAGAGGTTAGAGG	20

Supplementary Table S3. Putative G-quadruplex-forming sequences (PQSs) identified in the 3′ UTR regions of DENV-1 to DENV-4 using PQSfinder. Selected candidate PQSs for further analysis are highlighted.

Serotype	start	end	PQSfinder	G-score
DENV-1	10451	10472	**[GG][GG]ATGTAAAAACCT[GG]GA[GG]**	17
	10499	10523	[GG][GG]TAGCAGACTAGT[GG]TTAGA[GG]	14
	10553	10600	[GGGG]CCCAACACCA[GGGG]AAGCTGTACCCT[GGTGG]TAA[GGACTAGAGG]	43
DENV-2	10435	10458	**[GG]AA[GG]TGTAAAAAATCT[GG]GA[GG]**	15
	10485	10509	[GG]CGTAGT[GG]ACTAGC[GG]TTAGA[GG]	14
	10542	10555	[GG][GG]CCCAA[GG]T[GG]	29
	10576	10596	[GG]AA[GG]ACTAGA[GG]TTAGA[GG]	19
DENV-3	10414	10434	**[GG][GG]ACGTAAAGCCT[GG]GA[GG]**	19
DENV-4	10344	10373	**[GGG]A[GGCG]TAAAATTCCCA[GGG]A[GGCCATG]**	18
	10395	10419	[GG]CATATT[GG]ACTAGC[GG]TTAGA[GG]	14
	10458	10476	[GG][GG]CCCGAAGCCA[GG]A[GG]	21
	10490	10506	[GG]T[GG]AA[GG]ACTAGA[GG]	24

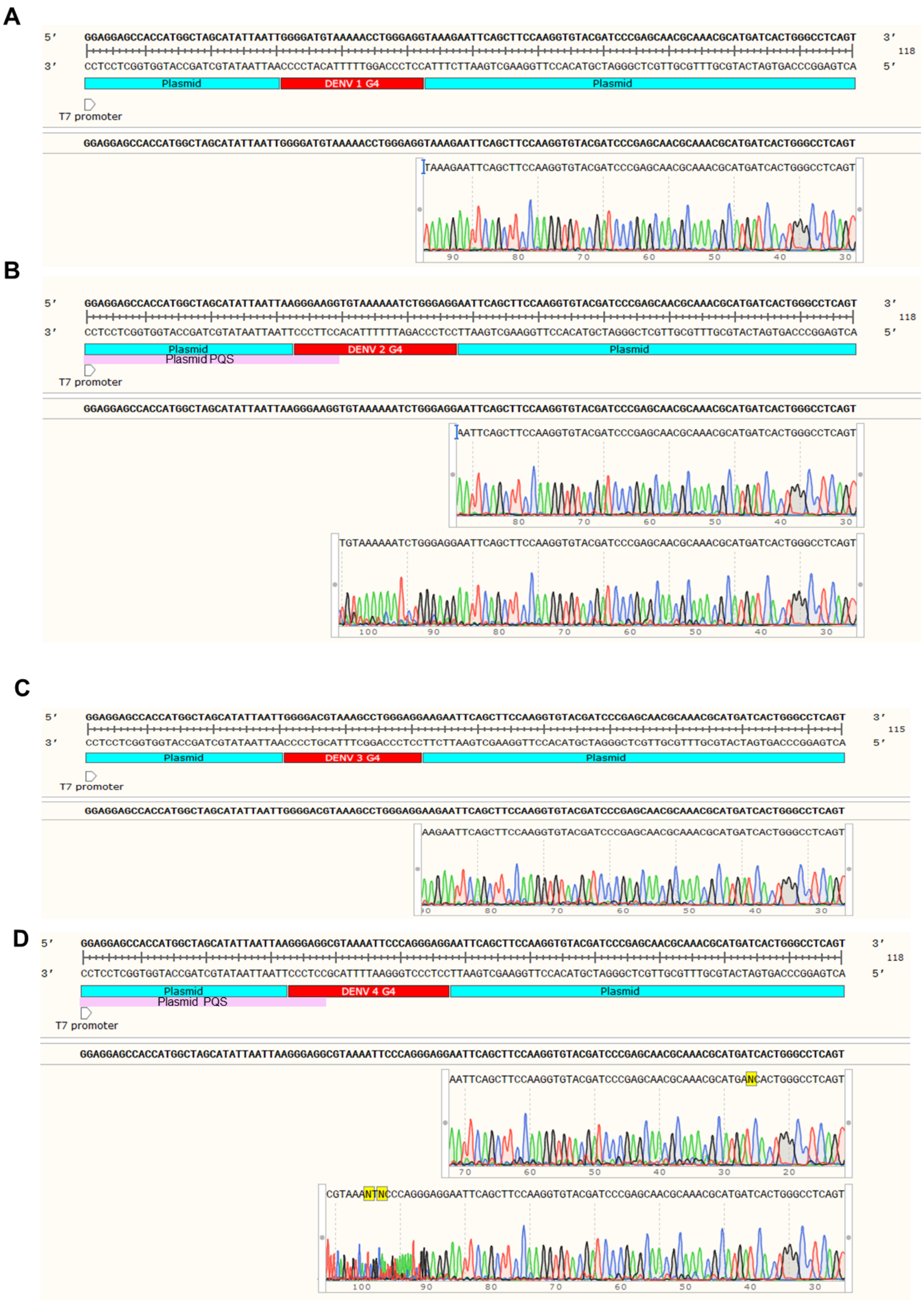

E

sequence	sequence parts	chromosome	fragment length	core_region	vienna RNA	centroid fold	mxfold2	predicted value
GGAGGAGCCACCATGGCTAGCATATTAATTAAGGGAAGG	,GG,A,GG,AGCCACCATGGCTAGCATATTAATTAA,GGG,AA,GG,	DENV 2	39	DENV 2 17-56	-5.59999 9905	-2.95	2.5	0.960538
GGAGGAGCCACCATGGCTAGCATATTAATTAAGGGAGG	,GG,A,GG,AGCCACCAT,GG,CTAGCATATTAATTAA,GGG,AGG	DENV 4	38	DENV 4 17-55	-5.59999 9905	-2.95	2.4	0.824726

Supplementary Figure S6. Sequence verification of RT-stop mapping. **(A-D)** Sanger sequencing mapping of RT-stop sites in **(A)** DENV-1, **(B)** DENV-2, **(C)** DENV-3 and **(D)** DENV-4 showing arrest at the G4-forming regions, In the construct maps, the red regions indicate the inserted DENV G4-forming sequences, the blue regions indicate plasmid-derived sequences, and the pink region indicates an additional plasmid-derived PQS detected in the DENV-2 and 4 construct. **(E)** Local predictor output showing the predicted G4-forming regions and prediction scores corresponding to the additional bands detected in DENV-2 and DENV-4.
