# Supplementary figures and images for: Identification of Ligand-Responsive RNA G-Quadruplexes in the 3′ UTRs of Dengue Virus Serotypes
Source: Biomolecules. 2026 Jun 25;16(7):946. doi: 10.3390/biom16070946 (PMC13407133; doi:10.3390/biom16070946)

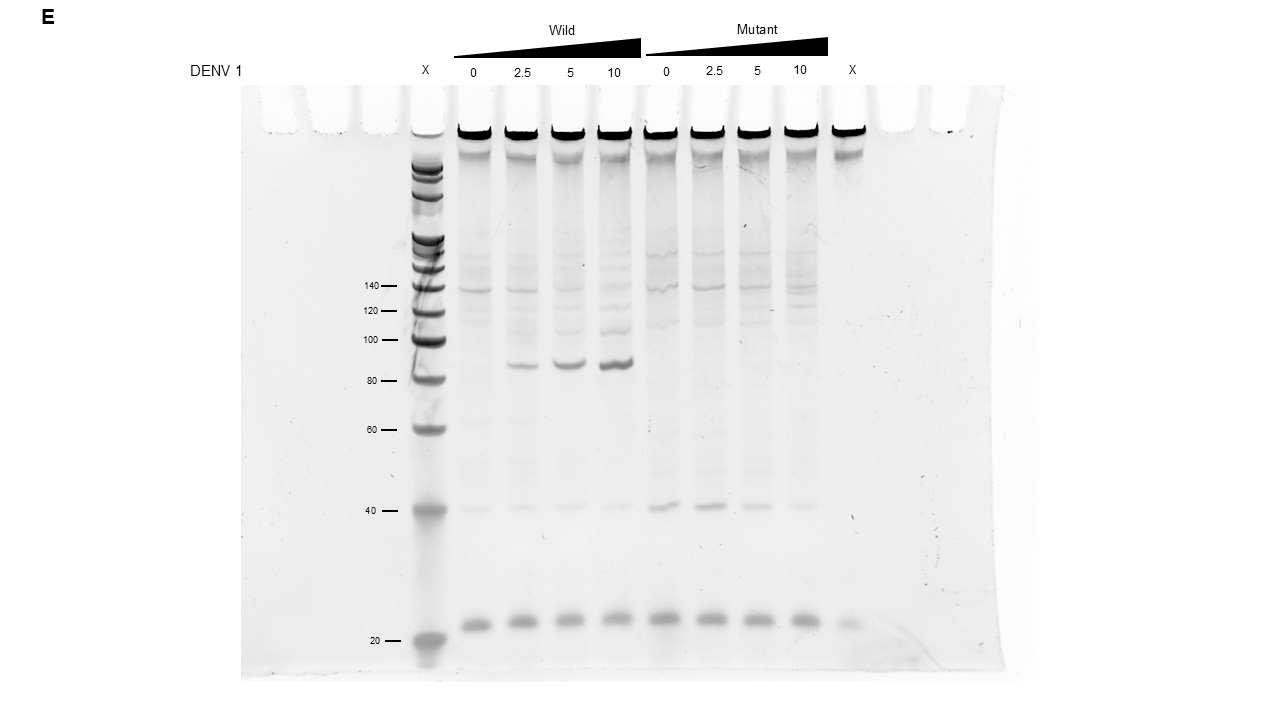

Supplement: Supplementary file 1 [file biomolecules-16-00946-s001.zip › DENV 1.tif]

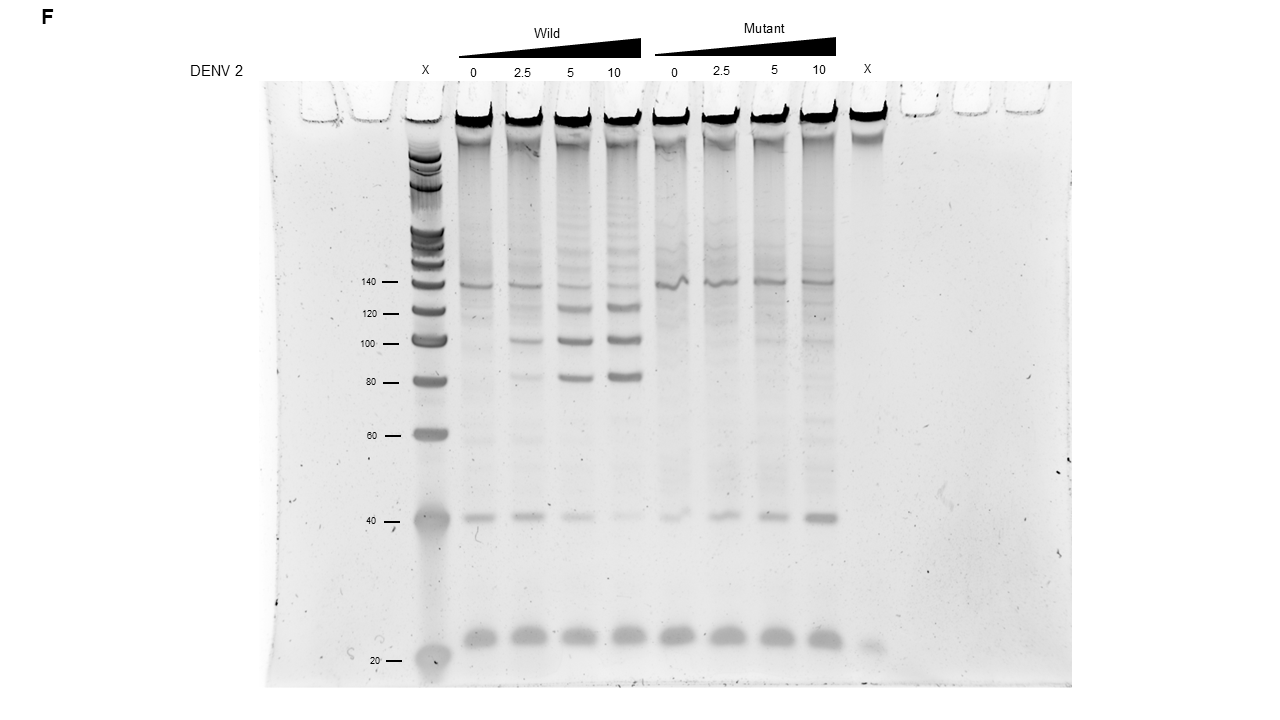

Supplement: Supplementary file 1 [file biomolecules-16-00946-s001.zip › DENV 2.tif]

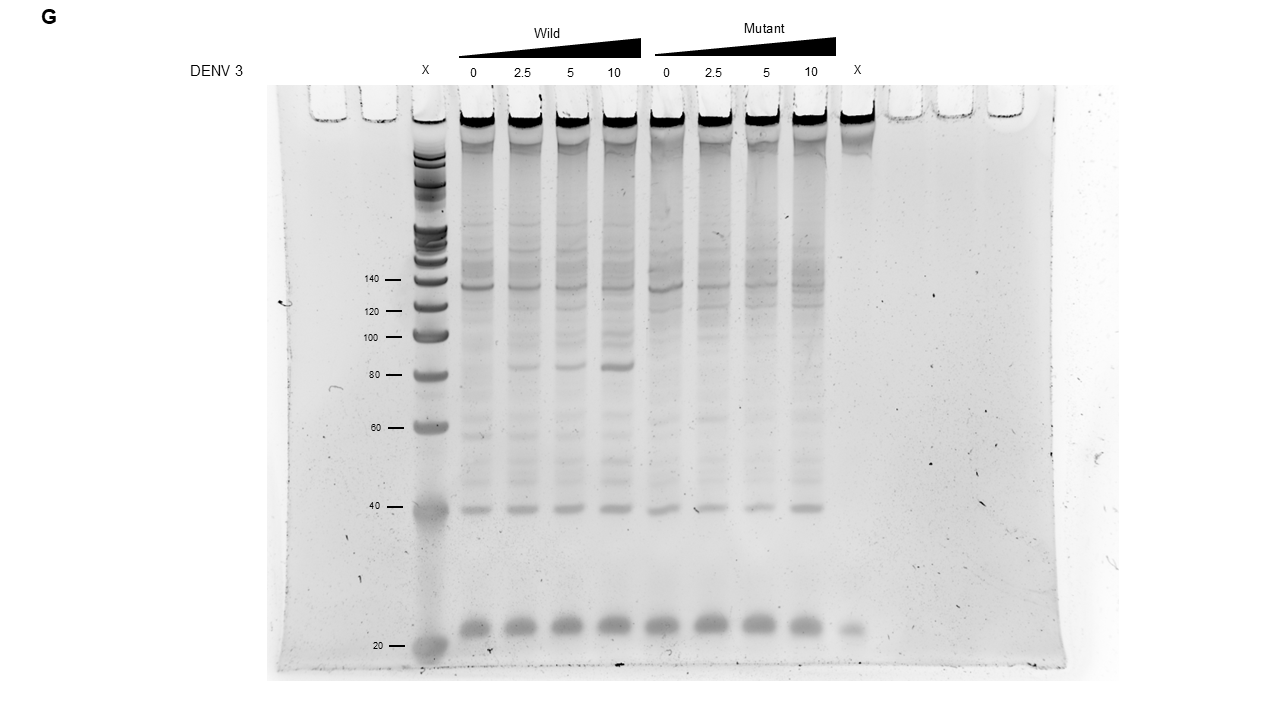

Supplement: Supplementary file 1 [file biomolecules-16-00946-s001.zip › DENV 3.tif]

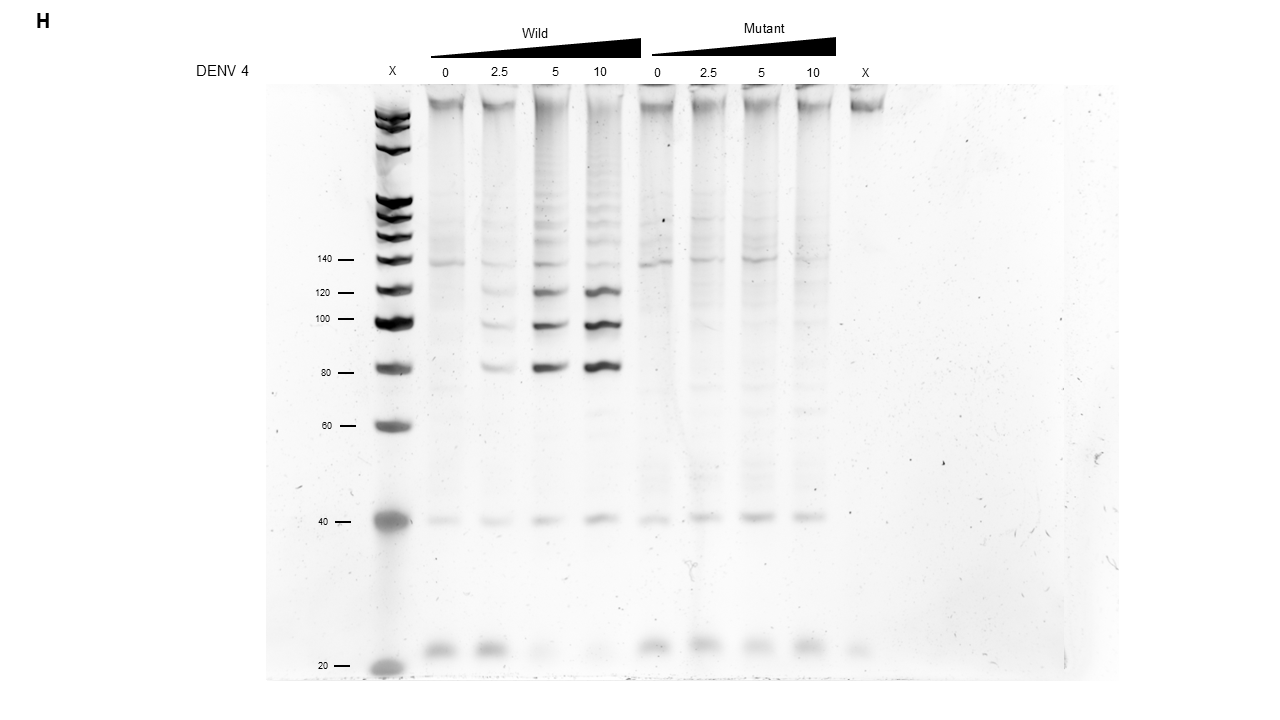

Supplement: Supplementary file 1 [file biomolecules-16-00946-s001.zip › DENV 4.tif]
